# An Artificial Intelligence System to Predict Quality of Service in Banking Organizations

**DOI:** 10.1155/2016/9139380

**Published:** 2016-05-22

**Authors:** Mauro Castelli, Luca Manzoni, Aleš Popovič

**Affiliations:** ^1^NOVA IMS, Universidade Nova de Lisboa, Rua de Campolide, 1070-312 Lisboa, Portugal; ^2^DISCo, Università degli Studi di Milano-Bicocca, Viale Sarca 336, 20126 Milan, Italy; ^3^Faculty of Economics, University of Ljubljana, Kardeljeva Ploscad 17, 1000 Ljubljana, Slovenia

## Abstract

Quality of service, that is, the waiting time that customers must endure in order to receive a service, is a critical performance aspect in private and public service organizations. Providing good service quality is particularly important in highly competitive sectors where similar services exist. In this paper, focusing on banking sector, we propose an artificial intelligence system for building a model for the prediction of service quality. While the traditional approach used for building analytical models relies on theories and assumptions about the problem at hand, we propose a novel approach for learning models from actual data. Thus, the proposed approach is not biased by the knowledge that experts may have about the problem, but it is completely based on the available data. The system is based on a recently defined variant of genetic programming that allows practitioners to include the concept of semantics in the search process. This will have beneficial effects on the search process and will produce analytical models that are based only on the data and not on domain-dependent knowledge.

## 1. Introduction

In recent years, the banking sector has faced a world-wide economic crisis that has led, especially in certain countries, to a restructuring of the banking industry [1]. In addition, the banking sector has been heavily hit by loss of confidence by both private and industrial customers. Thus, banks find it particularly difficult to attract new customers in today's challenging business environments. Considering these aspects, it is essential to ensure the satisfaction of existing customers in order to strengthen the credibility of the bank, to increase the loyalty of the customers, as well as preventing potential customer attrition. This is even more important in a sector where many competitors can offer the same services at similar conditions and where competition is particularly strong. Under this perspective, the quality of service provided to the customers is crucial. The concept of quality of service has been considered in a plethora of studies [2–4] and there is a large amount of work highlighting the importance of service quality for business performance in different sectors [5–7]. In this work we consider a narrower aspect of service quality, namely, the waiting time that customers must endure in order to receive a service. This is an important part of the overall service experience as improving the customer experience through shorter waiting times has positive effects on long-term customer loyalty [8].

In the context of a commercial bank, this issue is tightly linked to the management of the queues and the decision about the number of opened counters available to customers at a given time. This is a fundamental problem that bank managers must address and, in particular, the problem should be considered under two aspects: while it is important to ensure an adequate level of service that results in acceptable waiting times for customers, it is also important to minimize the number of opened bank counters in order to minimize operating costs. Obviously, the two requirements are diametrically opposed: from the point of view of the customer the best scenario involves opening all the existing counters in order to reduce waiting times, but this is clearly infeasible (or at least nonoptimal) from the point of view of the bank management because of the cost associated with this policy.

To bridge this gap, we propose a predictive model that relies on customer and transaction data and is able to predict the quality of service expressed as the waiting time that customers must endure before receiving a service. Based on this prediction, it will be possible for the bank managers to determine the number of counters required to satisfy customer demands while minimizing operating costs. The problem is of fundamental importance from the point of view of bank management as the cost associated with operating personnel is one of the most relevant items for the total operational costs. Clearly, the model must be as accurate as possible: overestimating the target level of quality of service results in additional costs for the bank, while a prediction that underestimates the quality of service will have a negative impact on customer loyalty. What is more, it may cause a decrease in the number of transactions because customers are not willing to wait a long time to receive the sought services.

To tackle this problem, we propose a system that involves the use of an artificial intelligence technique. There are several reasons to adopt such a system in the context of our study: while several methods based on standard statistical techniques exist, they are not able to produce a good model for the problem at hand. In fact, traditional methods are not the best choice for modeling phenomena characterized by a high variance. For instance, in the considered problem, the volume of customers that need a particular service can vary depending on the particular period of the year: usually before holidays the demand for banking services increases and the same happens before deadline for payments. On the other hand, during summer time the request for banking services usually decreases due to the closure of offices or commercial activities. These are just examples, but a plethora of events able to affect the request of banking services exists.

The use of an artificial intelligence technique can counteract the limits of standard statistical models. While the standard approach used for building analytical models relies on theories and assumptions about the applicative domain, in this work, we want to learn about a model just considering the available data. In order to do that, we consider genetic programming (GP) [9]. GP has been used to solve several real-life problems [10] and it is particularly suitable for addressing symbolic regression problem. Nevertheless, in its standard form, GP builds analytical models combining candidate solutions based on their syntax. That is, in its standard form GP completely ignores the information about employed data. This is probably one of the most important limitations for a wider diffusion and applicability of GP as it is challenging for a non-GP expert to trust a model built without considering the available data. This limitation has been addressed in the work of [11], where a GP version that considers semantic information has been proposed. The concept of semantics is closely related to the data and it allows GP to produce better models with respect to its standard, syntax-based version [12, 13].

The remainder of the paper is organized as follows: Section 2 describes the standard GP algorithm and the operators used in the search process.  Section 3 defines the concept of semantics and presents the semantic operators used in this paper. More specifically, we highlight the benefits of the semantic operators on the search process.  Section 4 describes the experimental phase and discusses the obtained results.  Section 5 provides the main take-away from our work while suggesting some avenues for future research.

## 2. Genetic Programming

**Figure 1:**
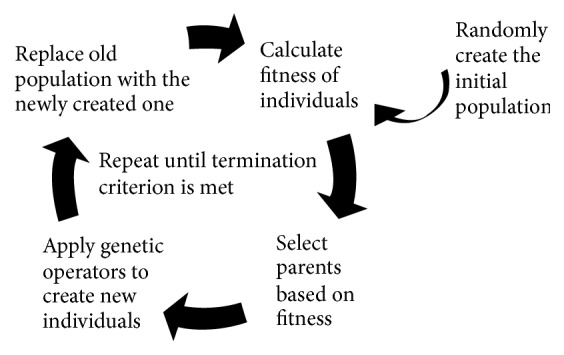
The genetic programming algorithm.

GP belongs to the family of bioinspired computational intelligence techniques. GP is used for obtaining optimal (or suboptimal but still near the optimum) solutions to optimization problems. The main idea of GP is to mimic the biological evolutionary process in order to create, iteration by iteration, better solutions to the problem at hand. The GP search process is reported in Figure 1.

In GP candidate solutions are represented using a Lisp-like tree structure. In order to create new solutions, GP makes use of stochastic operators called genetic operators. These operators are crossover and mutation. In the standard version of GP, these two operators work as follows: given two solutions (called parents solutions), crossover builds two new solutions by swapping a subtree of the first parent with a subtree of the second parent. The subtrees are randomly chosen. Mutation only acts on one solution and it is used to introduce some variation on the incumbent solution: in particular, given a tree, mutation creates a new solution by replacing a randomly chosen subtree with a newly generated subtree that has a maximum allowable depth.

As it can be observed, these operators act on the structure (i.e., the syntax) of the individuals and they ignore the information related to the semantics of the solutions. From now on, we refer with the term semantics to the vector of outputs produced by a candidate solution considering the set of training instances. While the definition of semantics is not unique, this is the definition of semantics that is typically used [14, 15].

Since its definition, GP has been used to solve complex problems in several domains only considering syntax-based genetic operators. There are several reasons for this. To begin with, abstraction from semantics allows GP to use simple genetic operators that are easy to define and that are independent from the particular application at hand. Hence, standard genetic operators can be used for addressing regression, classification, or even clustering problems without changing their definition. Next, a solid theory exists that guarantees the convergence of standard GP towards optimal solutions [16]. This theory has been formulated considering standard, syntax-based genetic operators. Nevertheless, relying on syntax-based genetic operators results in some difficulties. The main drawback is that the abstraction from semantics will produce solutions that completely ignore the knowledge associated with the available data. The second drawback, which is actually one of the main causes that has limited the use of GP outside the artificial intelligence community, is that it is difficult for an expert of a particular domain to accept as a solution to a particular problem an analytical model built without considering the available data. That is, in its standard form, GP only uses data to calculate the value of the objective function (also called fitness function) that quantifies the quality of a candidate solution.

To counteract all these limitations, research has recently focused on the definition of methods that are able to consider the semantic information in the search process. The next section will introduce the main concepts related to the definition of semantic-based methods, describing the semantic genetic operators that have been used in this paper.

## 3. Semantic Operators

In order to offset the limitations of standard GP pointed out in the previous section and also with the aim of improving its performance, research in the field of GP has lately focused on the definition of methods based on the concept of semantics. A large number of works have been proposed [17] and, while the definition of semantics is not unique, it is common to refer with this term to the vector of output produced by a GP individual considering a set of training instances.

Nowadays it is possible to distinguish two different ways of incorporating semantics in the GP process: through direct methods and through indirect methods. Indirect methods represent the first attempt of including semantic information in GP and they basically share the following idea: the GP algorithm makes use of standard genetic operators, yet, after the creation of a new candidate solution, a test based on the semantics of the newly created solution is performed. If the new solution satisfies the semantic criteria then it is accepted, while in the opposite case the newly created individual is rejected. Hence, semantics is included indirectly in the search process. While this idea allows researchers to include semantics information in GP in a very simple way, it results in an unacceptable overhead in terms of time, with a large number of rejected individuals. The problem is even more relevant considering that indirect methods usually require calculating the fitness of the individuals (a time consuming task) even if they are rejected. To overcome the limits of indirect methods, research has been focused on methods able to directly include the semantic information in the search process. The work presented in [11] describes such idea: instead of using traditional syntax-based genetic operators, authors of [11] have defined new genetic operators that directly act on the semantics of the candidate solutions. This allows GP to create solutions that present particular semantic features, thus removing the task of verifying the respect of semantic criteria.

The inclusion of semantic information by means of the genetic operators proposed in [11] presents several advantages with respect to the use of standard GP: it has been proved that semantic-based operators are able induce a unimodal fitness landscape [18] on any problem consisting in finding the match between a set of input data and a set of expected target ones (like regression and classification problems). According to the theory of the fitness landscapes this will increase GP evolvability, which is the ability of GP in finding better quality solutions. Considering the business analytics perspective, another advantage is also evident: moving from standard GP (where the search process ignores the semantic information) to a semantic-based GP allows practitioners to take advantage of the large amount of data available today. That is, diversely from standard GP, semantic GP exploits the concept of data-driven framework.

In this paper, we will consider the definition of semantic operators for real functions domains, since these are the operators that we will use in the experimental phase. The definitions of semantic crossover and semantic mutation are the following.

*Semantic Crossover.* Given two parent functions *T*_1_, *T*_2_: *ℝ* ^*n*^ → *ℝ*, semantic crossover returns the real function *T*_*XO*_ = (*T*_1_ · *T*_*R*_)+((1 − *T*_*R*_) · *T*_2_), where *T*_*R*_ is a random real function whose output values range in the interval [0,1].

To constrain *T*_*R*_ in producing values in [0,1] we use the sigmoid function *T*_*R*_ = 1/(1 + *e* ^−*T*_rand_^), where *T*_rand_ is a random tree with no constraints on the output values. 

*Semantic Mutation.* Given a parent function *T*: *ℝ* ^*n*^ → *ℝ*, semantic mutation with mutation step ms returns the real function *T*_*M*_ = *T* + ms · (*T*_*R*1_ − *T*_*R*2_), where *T*_*R*1_ and *T*_*R*2_ are random real functions with codomain in the range [0,1].

Reference [11] formally proves that this operator corresponds to a box mutation on the semantic space and induces a unimodal fitness landscape.

While these operators have several advantages (reported in [11]) with respect to the standard ones, there is an important limitation that must be considered. As it can be easily noticed considering their definition, every application of these operators produces an offspring that contains the complete structure of the parents, plus one or more random trees as its subtrees, and some arithmetic operators: the size of each offspring is thus clearly much larger than the one of their parents. In order to counteract this exponential growth of the individuals [11] that makes it difficult to use these operators for addressing real-life problems, in this paper we use the solution proposed in [15]. More in detail, the work described in [15] proposed a very simple and effective implementation of the GP algorithm that allows GP to use the semantic operators in a feasible way. This is the implementation used in this paper and also documented in [19].

## 4. Experiments

This section describes the problem that has been considered, the employed data, the experimental settings, and the obtained results.

### 4.1. Problem and Data

As reported in the previous sections, the objective of the proposed GP system is to build a model that is able to predict the quality of service. We want to give to bank managers a tool to determine the minimum number of bank counters to open in order to satisfy customers' requests in an acceptable amount of time. In order to build an analytical model able to predict the quality of service, we considered historical data related to transactions of several banks. In particular, each record associated with a particular transaction consists of several pieces of information: type of requested service, number of customers in the bank, volume of transactions completed in that day, and additional information related to the customer account. Finally, there is a set of information collected from a survey filled by customers. The survey contains information related to the perceived quality of service and can be filled by the customers in any moment. That is, a customer can give his feedback also without having been served. That is the case, for instance, in which the customer leaves the bank because the waiting time is too long and no enough counters are opened. It is important to take into account this last set of information related to the perceived quality of service: a waiting time that is acceptable for a particular customer can be perceived as not acceptable by another customer.

To each transaction is associated a target value that represents the quality of service expressed as the waiting time a customer has to endure before being served (or before deciding to leave the bank). Target values are in the range [0,1] because the provided dataset (available at http://www.cs.toronto.edu/~delve/data/datasets.html) has been normalized.

The considered dataset contains more than 8 thousand transaction records, and each record consists of a set of 32 real-value attributes.

### 4.2. Experimental Settings

This section reports the experimental settings. We will refer to the proposed system with the term GS-GP (geometric semantic genetic programming). We perform 30 independent runs of GS-GP and, in each run, we consider a different partition of the dataset. In detail, 70% of the available transaction records are used for building the analytical model, while the remaining 30% are used for testing the system on unseen data, hence evaluating its generalization ability. The execution of 30 runs is a fundamental aspect considering the stochastic nature of GP.

We compare the performance of the proposed system with the one of standard GP (ST-GP). This comparison is performed for two main reasons: first of all we want to evaluate if the introduction of the concept of semantics in the search process has any beneficial effect and, secondly, we want to discuss the differences in terms of generalization of the two considered systems. This last point is particularly important for managers: having a model that performs poorly on the unseen data is completely useless in a real scenario, independently of the results achieved in the training phase.

All the runs use a population of 200 individuals and the evolution ends after 1,000 generations. The function set contains the four binary arithmetic operators +, −, *∗*, and / protected as in [9]. Fitness is calculated as the root mean square error (RMSE) between predicted and target values. Trees initialization is performed with the Ramped Half-and-Half method [9] with a maximum initial depth equal to 6.

The terminal set contains 32 variables, each one corresponding to a different feature in the dataset and 50 random constants in the range [−10,10]. To create new individuals, ST-GP uses standard (subtree swapping) crossover [9] and (subtree) mutation [9]. Crossover and mutation probabilities are equal to 0.9 and 0.1, respectively. For GS-GP, a mutation step equal to 0.1 is used. These values have been chosen after a preliminary tuning phase, in which different values of the parameters have been tested. We finally selected the combination that returned the best results. Anyway, it is interesting to point out that, in many cases, the differences between the tested combinations were not statistically significant: in other words, it appears that the parameter setting does not significantly influence the performance of GS-GP and ST-GP, at least for the considered application. Survival from one generation to the other is always guaranteed to the best individual (elitism). No maximum tree depth limit is imposed during the evolution.

### 4.3. Results

The comparison between ST-GP and GS-GP is performed according to three different criteria: the fitness on the training set, that is, the ability of the algorithm to learn the data, the fitness on the test set, that is, the ability to correctly predict the output on unseen data, and the time needed to generate the model. As we can observe, GS-GP outperforms ST-GP on all the considered criteria. To compare the results, a rank-based statistical test has been used. More in detail, the Mann-Whitney *U* test with a sensitivity of *α* = 0.05 has been considered, with the alternative hypothesis that GS-GP produces better results than ST-GP with probability greater than one half.

**Figure 2:**
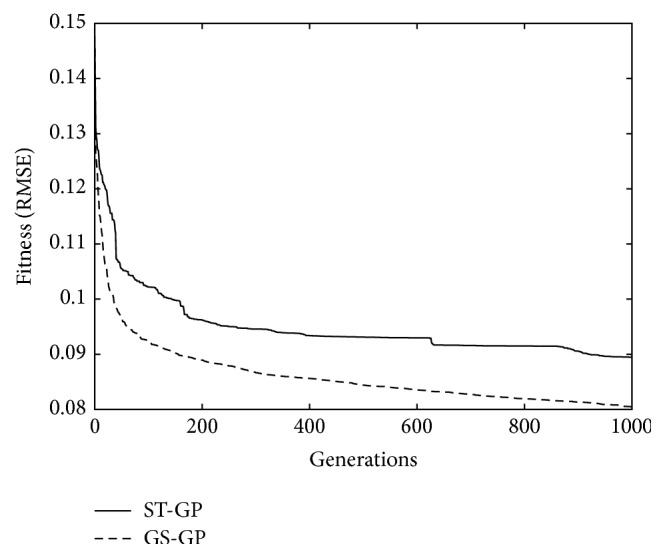
Training fitness. The curves report, at each generation, the median of the fitness of the best individual in the population, computed over the 30 independent runs of the algorithm.

The first comparison between ST-GP and GS-GP is shown in Figure 2, where the median of the training fitness of the best individual in the population, generation by generation, is presented for the two considered techniques. As it is possible to observe, in every generation, GS-GP performs better, that is, has lower error, than ST-GP. It is also interesting to analyze how the fitness changes: for ST-GP the decrease of the error is not gradual, with a fitness that gets stuck on a certain value for a large number of generations and that quickly decreases before getting stuck again (this is particularly clear between generations 400 and 600). On the other hand, the decrease of the error of GS-GP is more consistent across generations. This behavior allows us to predict the decrease of the error and to stop the evolution accordingly, a task that is not easy to perform for ST-GP.

**Figure 3:**
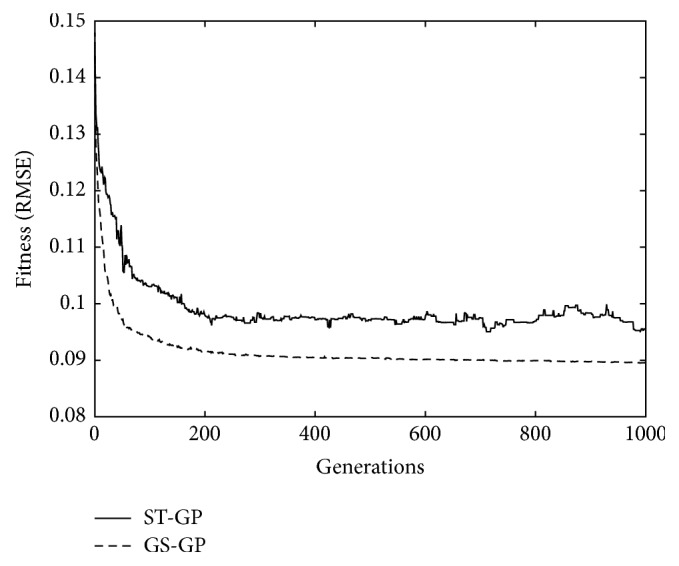
Test fitness. The curves report, at each generation, the median of the fitness of the best individual in the population, computed over 30 independent runs.

Figure 3 provides a second comparison between GS-GP and ST-GP, by plotting, for each generation, the median, over the 30 runs, of the fitness obtained on the test set by the best individual (as determined by the results obtained on the training set). These results are interesting since they provide an indication of the ability of the obtained model to correctly predict the output on unseen data. The behavior of the two algorithms is greatly different. ST-GP has a decrease of the error that continues up to generation 200. After this point, the error on unseen data continues to oscillate and, before generation 1000, it even increases to more than 0.1. On the other hand, for GS-GP, there is a more predictable behavior: there is a noticeable decrease of the error up to generation 200 and, after that, there is still a decrease, only less pronounced. There are no oscillations and better results on the training set do not translate to a worst generalization ability. Even in this case, GS-GP performs better than ST-GP.

**Figure 4:**
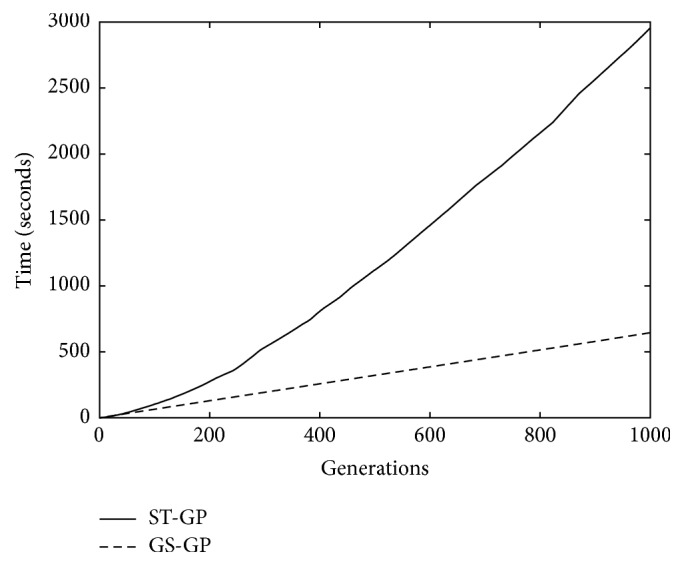
The median time (in seconds) needed for ST-GP and for GS-GP.

The third comparison is reported in Figure 4, where the median time needed to reach the *i*th generation (for 0 ≤ *i* ≤ 1000) is presented. This comparison is not directly related to the goodness of the obtained model, but it is useful to perform a choice when there are two or more available methods and the time needed to generate a model is one of the criteria for selecting one of the methods. In the previous two comparisons, we have observed that GS-GP performs better than ST-GP. Here, we show that not only is GS-GP able to produce better results with respect to ST-GP, but the time needed to do this is also lower. The reason relies on the different versions of GP under exam. While ST-GP uses genetic operators that act on the syntax of the solutions, GS-GP only considers semantic information (i.e., strictly related with the available data). This difference results in the possibility of running GS-GP in a very efficient way: the current implementation of GS-GP [15] requires a constant time for each generation, as it is possible to see in the plot. On the other hand, in all the existing implementations of ST-GP, the time needed to execute all the steps that comprise a generation depends on the structure of the trees that are in the current population. In particular, it has been observed that, generation after generation, the average size of the trees (i.e., the number of nodes) in ST-GP usually increases. This causes a slowdown in the execution of the ST-GP algorithm in later generations, as it is possible to observe in the plot. As a conclusion, even when the time is considered as a comparison criterion, ST-GP is outperformed by GS-GP.

**Figure 5:**
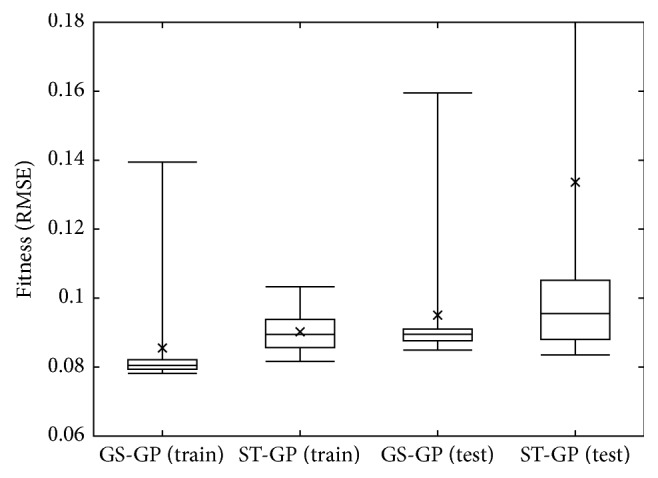
Boxplots of the fitness obtained at the last (1000th) generation.

The comparison of the results obtained in the last generation is summarized in the boxplot of Figure 5. The boxes represent the 25th and 75th percentile, the central bar represents the median, the two whiskers represent the maximum and the minimum obtained, and the cross represents the average. This plot corroborates the observation made when looking at Figures 2 and 3. In particular, it is possible to observe that GS-GP produces more consistent results with respect to ST-GP, as evinced by the size of the boxes.

To analyze the statistical significance of these results, a set of tests has been performed on the median errors. In particular, we want to assess whether the final results (generation 1000), produced by the two systems, have a statistically significant difference. As a first step, the Shapiro-Wilk test (with *α* = 0.1) has shown that the data are not normally distributed and hence the nonparametric Friedman test has been used. The null hypothesis for the comparison across repeated measures is that the distributions (whatever they are) share the same median. The alternative hypothesis is that distributions across repeated measures have unequal medians. Also for this test a value of *α* = 0.1 has been considered.

The statistical tests confirm that, in all the comparisons, GS-GP outperforms ST-GP. In particular, considering the training fitness, the statistical test gives a *p* value of 5.3 · 10^−7^, on the test fitness a *p* value of 0.033, and, with respect to the execution time, a *p* value of 1.4 · 10^−11^. Hence, it is possible to conclude that, for the considered application, the best performer is GS-GP and there is no motivation (performance, generalization ability, or time) to choose ST-GP to address the problem at hand.

### 4.4. Comparison with Other Techniques

Besides comparing GS-GP against ST-GP, it is also important to compare the performance of GS-GP against other well-known methods. In particular, we take into account least square regression (SQ), radial basis function network (RBF), multilayer perceptron (MLP), and isotonic regression (ISO).

Table 1 reports the values of the training and test errors (RMSE) of the solutions obtained by all the studied techniques including, in the last rows of the table, GS-GP. For all the considered techniques, we used the implementation available in the WEKA machine learning tool [20]. Moreover, we used the functions provided by WEKA for finding the best parameter settings for the techniques taken into account. In particular, the tuning phase has been performed by using the WEKA metaclassifier (CVParameterSelection). The metaclassifier provides a way of automating the tuning process.

From these results, it is possible to see that GS-GP is the best performer on test instances, followed by ISO. MLP is the worst performer on test instances. Considering training fitness, MLP is the best technique, followed by GS-GP and ISO. It is interesting to point out how MLP overfits training data, while GS-GP does not present this undesirable phenomenon. The worst performer on training instances is square regression that achieves also on the test instances the same deprived performance.

To assess the statistical significance of these results, the same set of tests described in the previous section has been performed. In this case, a Bonferroni correction for the value of *α* has been considered, given that the number of compared techniques is larger than two. All the *p* values relative to the comparison between GS-GP and the other methods are reported in Table 2. According to the results reported in the table, the differences in terms of training and test fitness between GS-GP and all the other considered techniques are statistically significant. These results confirm the appropriateness of the proposed method for addressing the problem at hand.

## 5. Conclusions

In this paper we propose an artificial intelligence system for predicting the quality of service of a bank. The quality of service has been considered as the waiting time that the user must endure before being served. Based on the current level of quality of service, managers can decide to open additional bank counters in order to satisfy customers' requests. The application is particularly important: while offering a good quality of service will increase customers' loyalty, it is also important to reduce the operating costs associated with the opened bank counters. Hence, under the managerial perspective, it is important to find the compromise that allows guaranteeing a good service quality while keeping operating costs low.

The application of an artificial intelligence technique tries to overcome the limitations of traditional statistic based linear regression methods. The main problem is that these techniques are unable to adapt to unusual circumstances, which form a highly nonlinear relationship with customers' requests. Hence, their predictions are not as satisfactory as desired.

While the usage of genetic programming allows practitioners to discover analytical models without any knowledge related to the applicative domain, in its standard form GP also ignores the information associated with the available data. To overcome this limitation, in this work, we used recently defined genetic operators that allow GP to include the concept of semantics in the search process. These operators have several advantages with respect to the traditional ones; in particular, they are able to induce a unimodal fitness landscape in all the problems that consist in finding a match between target and obtained values (i.e., like regression problems).

Experimental results have shown the suitability of the proposed method for predicting the quality of service. In particular, not only does the proposed system outperform standard GP and other well-known techniques, but it is able to produce models that generalize better on unseen instances. Moreover, the time required by the proposed system for building a model is significantly lower than the one required by standard GP. This is particularly important considering the large amount of historical transactional data that are nowadays available.

## Figures and Tables

**Table 1:** Experimental comparison between different nonevolutionary techniques and GS-GP. Median of the training error and test error (RMSE) calculated over 30 independent runs.

Method	Training error	Test error
Least square regression [21]	0.127	0.126
Radial basis function network [22]	0.122	0.121
Multilayer perceptron [22]	0.068	0.138
Isotonic regression [23]	0.103	0.103
GS-GP	0.0908	0.0924

**Table 2:** *p* values obtained from the statistical validation procedure.

		SQ	RBF	MLP	ISO
GS-GP	TRAIN	8.40*E* − 09	8.45*E* − 09	1.69*E* − 09	1.06*E* − 04
	TEST	2.83*E* − 08	1.07*E* − 07	5.00*E* − 09	1.06*E* − 07
